# Aerobic training and hydroalcoholic extracts of green tea improve pro-oxidant-antioxidant balance and histopathological score in the N-methyl-N-nitrosourea-induced prostate cancer model of rat

**DOI:** 10.17179/excli2019-2069

**Published:** 2020-06-08

**Authors:** Zakaria Vahabzadeh, Mohammadraman Molodi, Bahram Nikkho, Marziyeh Saghebjoo, Saber Saedmocheshi, Fatemeh Zamani, Yazdan Roshani, Sina Babanzadeh

**Affiliations:** 1 Liver & Digestive Research Center, Research Institute for Health Development, Kurdistan University of Medical Sciences, Sanandaj, Iran; 2 Department of Clinical Biochemistry, Faculty of Medicine, Kurdistan University of Medical Sciences, Sanandaj, Iran; 3 Cellular and Molecular Research Center, Research Institute for Health Development, Kurdistan University of Medical Sciences, Sanandaj, Iran; 4 Department of Physiology and Pharmacology, Kurdistan University of Medical Sciences, Sanandaj, Iran; 5 Department of Pathology and Clinical Laboratory Sciences, Kurdistan University of Medical Sciences, Sanandaj, Iran; 6 Department of Exercise Physiology, Faculty of Sports Sciences, University of Birjand, Birjand, Iran

**Keywords:** prostate cancer, pro-oxidant-antioxidant balance, prostate-specific antigen, Camellia sinensis, aerobic exercise

## Abstract

Green tea is a main resource for catechins. Catechins as antioxidant compounds reduce the production of reactive oxygen species and they have a protective role in the development of cancer. As well as, aerobic exercise change free radicals with two contradictory mechanisms. Aerobic training promotes excessive production of free radicals resulting in oxidative stress. In contrast, it increases the total antioxidant capacity. In this study, effect of aerobic training and hydroalcoholic extract of green tea (HEGT) on the pro-oxidant-antioxidant balance (PAB), prostate-specific antigen (PSA) and histopathological score of cancerous tissue in the N-methyl-N-nitrosourea-induced prostate cancer was investigated. A rat model of prostate cancer was induced by hormonal change and N-Nitroso-N-methylurea (NMU). HEGT 0.1 % and 45 min of aerobic exercise in 5 days a week for eight weeks were scheduled. The presence of major catechins was approved using GC-MS. Histological study, PAB and PSA levels were used to monitor the preventive role of treatments. The prostate weights of cancerous rats were significantly higher than healthy controls (P<0.05). The PAB was only significantly higher in cancerous rats and cancerous rats receiving aerobic exercise (P<0.05). The mean of histological score of cancerous tissue was reduced in rat receiving HEGT and both HEGT and aerobic exercise. The amounts of PSA level did not significantly differ between the groups of this study (P>0.05). Our results provided laboratory and histological documentation for the preventive role of green tea extract in developing prostate cancer via its potential to re-establish the pro-oxidant-antioxidant balance.

## Introduction

Cancer as multifactorial and very complex disease is still one of the leading causes of death and the most challenging human problem to overcome or prevent it (Cunningham and You, 2015[13]). In addition to chemotherapy, which is the main line of treatment for many cancers, a large number of studies have previously highlighted the beneficial effects of plant extract and exercise against different cancers including prostate cancer. Although several protective mechanisms have been proposed for each of these two mentioned cases, the exact/detailed mechanism is still unclear.

The green tea (*Camellia sinensis*) extract, like many other herbal extracts, has been noted to have numerous bioactive compounds including flavonoids, flavanols and phenolics acids which give anti-oxidant properties. Epicatechin (EC), epicatechin-3-gallate (ECG), epigallocatechin (EGC), and epigallocate-chin-3-gallate (EGCG) are the major polyphenols supposed to have anti-oxidant properties. Previous studies showed the ability for catechins to reduce the risk of prostate cancer through the affecting on the proliferation, angiogenesis and/or metastasis mainly because of their potential anti-oxidative effects (Graham, 1992[15]; Fujiki et al., 1998[14]; Naponelli et al., 2017[29]). 

Exercise has been showed to promote the hyperproduction of free radicals which lead to oxidative stress (Park and Kwak, 2016[31]). In other words, there are many other studies that emphasize the beneficial effects of aerobic exercise due to increase in the total antioxidant capacity. Aerobic exercise has been shown to decrease the risk of advanced prostate cancer and mortality (Nilsen et al., 2006[30]; Alibhai et al., 2018[2]).

Oxidative stress is a situation in which an imbalance between the production of free radicals and the total antioxidant defense system including both enzymatic and nonenzymatic antioxidants exists (Migdal and Serres, 2011[27]). Generally, oxidative stress leads to the production of reactive oxygen or nitrogen species which initiates or promotes the numerous problems in cancers or other diseases. Until now, the usual method for evaluating of oxidative stress was to measure total free radicals and total antioxidant capacity as two separate evaluations. Pro-oxidant-antioxidant balance (PAB) directly evaluates the mentioned imbalance in oxidative stress (Alamdari et al., 2007[1]). Therefore in this study, we examined the PAB, prostate-specific antigen (PSA) and histopathological score of cancerous tissue in the prostate cancer model of rat and fallowing their treatments with hydroalcoholic extracts of *Camellia sinensis* and/or aerobic training in different groups.

## Material and Methods

### Animals and experimental treatments 

Sixty adult male wistar rats aged 45 days with initial body weight of 250 ± 20 g were used for this study. After acclimatization, the animals were then randomly divided into six groups consisting of eight rats each, and were housed four per cage under standard conditions. The animals were then treated in this order: Healthy normal rats (CON group) only received a normal chow. In PC group, prostate cancer was induced in the animals. In the PC+GTE group, rats with induced prostate cancer received 0.1 % green tea extract. In the PC+EXR group, rats with induced prostate cancer were forced to perform an aerobic exercise. PC+GTE+EXR group animals with induced prostate cancer received 0.1 % green tea extract and simultaneously were forced to perform an aerobic exercise. In sham group, healthy normal rats received an injection of physiological saline solution as well as 1 ml of that solution as gavage. The animals were maintained in their respective groups according to the principles of national institutes of health's guide for the use and care of laboratory animals and approved by the ethic committee of Kurdistan University of Medical Sciences (Ir.muk.REC.1396.184) for a period of eight weeks. They had free access to the normal chow and water ad libitum during the study. Food and fluid intake and body weight were measured weekly. At the end of the experimental period, the rats were anesthetized with ketamine (50 mg/kg), and xylazin (4 mg/kg). Blood was collected by direct cardiac puncture in heparin tubes. Plasma was then separated in two aliquots after centrifugation at 5000 rpm for 2 min at 4 °C and stored at -80 °C until biochemical analysis. The prostate was also removed, weighted and quickly fixed in the Bouin's solution for histological studies.

### Prostate cancer induction protocol

According to the previous studies to induce the prostate cancer in the cancerous groups, the animals received a daily dose (50 mg/kg body weight) of cyproterone acetate (Sigma-Aldrich, St Louis, MO, USA) as intraperitoneal injections for 18 consecutive days. Cyproterone acetate (CA) was used to prevent release of androgens from testis and to induce atrophy in the prostate epithelium. On day after the last injection of CA, the animals received a daily subcutaneous injection (100 mg/kg) of testosterone propionate (Sigma-Aldrich, St Louis, MO, USA) for three days to stimulate proliferation of prostate epithelium. On day after the last injection, the N-Methyl-N-nitrosourea (NMU, Sigma-Aldrich, St Louis, MO, USA) was injected to the animals as intraperitoneal at a dose of 50 mg/kg. The NMU powder was first wetted with 3 % acetic acid and then diluted with saline to prepare a final concentration 10 mg/mL with pH 5.5 for injection (Bosland and Prinsen, 1990[7]; Arroyo-Acevedo et al., 2017[3]).

### Histopathological studies of prostate

To confirm the histological changes of prostate in the cancerous models as well as among the different treated groups of animals, the dorsal and ventral lobes of prostate were quickly fixed in the Bouin's solution and consequently embedded in paraffin. Then, the 5-µm thick sections were cut from the paraffin-embedded tissue, mounted on slides, and stained with hematoxylin and eosin (H&E). To evaluate the histological changes and proliferative lesions of prostate in the animals, the histological examination of tissues were monitored by a pathologist according the histological grading scheme used in the previous studies (Suttie et al., 2003[38]).

### Preparation of hydroalcoholic extracts of green tea 

Briefly, 250 ml of 80 % methanol was added to 250 g of powdered green tea leaves (*Camellia sinensis*) and incubated at 28 °C for 24 h. Then, the extract was filtered by Whatman filter NO.1 and dried by a rotary evaporator under vacuum pressure. The remaining materials were resuspended in the water to prepare a solution of 0.1 % green tea (Gupta et al., 2001[17]; Siddiqui et al., 2008[37]). GC-MS analysis of the final solution was done to confirm the presence of epigallocatechin gallate (EGCG), epigallocatechin (EGC), epicatechin gallate (ECG), and epicatechin (EC) as the four major polyphenolic catechins (Saedmocheshi et al., 2019[34]).

### Aerobic exercise protocol

During this study, the animals received an eight-week aerobic exercise in their appropriate groups. They were forced to run on the treadmill for 5 days a week. They ran for 45 minutes every day in three 15-minute episodes with two minutes of rest between each. The intensity of given exercise was gradually increased from 3 m/min to 10 m/min. This intensity is equal to 60 % of maximal aerobic capacity for animals and it is well tolerated by the animals (Musch et al., 2004[28]). Moreover, the previous studies showed that this amount of aerobic exercise is enough to change the tumor progress (Cohen et al., 1992[12]).

### Determination of prostate-specific antigen

The amount of prostate-specific antigen (PSA) was measured in the plasma obtained from animals by an appropriate ELISA which was specific for prostate specific antigen of rat according to its manual (ZellBio, Germany).

### Determination of pro-oxidant-antioxidant balance 

A previously described colorimetric method was used to measure the pro-oxidant-antioxidant balance (PAB) (Sahebari et al., 2015[35]). Briefly, five different standard solutions (0-100 %) were prepared by mixing of 250 μM hydrogen peroxide with 3 mM uric acid (in 10 mM NaOH). Ten microliters of each sample, standard or blank (distilled water) were mixed with 200 µL of working solution containing 1 mL TMB cation with 10 mL of TMB solution, in a well of a 96 well plate. The plate was then incubated in a dark place at 37 °C for 12 min finally; 100 µL of 2N HCl was added to each well; and the absorbance was measured at 450 nm with a reference wavelength of 620 nm. For preparation of TMB solution, 200 μL of TMB/DMSO (60 mg TMB in 10 ml DMSO) was added into 10 mL of acetate buffer [0.05 M buffer, pH 5.8]. To prepare the TMB cation, 400 μL of TMB/DMSO, 70 μL of fresh chloramine T (100 mM) and 25 U of peroxidase enzyme were added into 20 mL of acetate buffer [0.05 M buffer, pH 4.5] at room temperature and in a dark place. The values of PAB which represent the percentage of hydrogen peroxide were calculated using the prepared standard curve and expressed in arbitrary units in the unknown samples.

### Statistical analysis

Statistical analysis was performed using the SPSS software (version 20). All values are presented as mean ± standard error of the mean. One-Way ANOVA with post-hoc Tukey test was performed to compare the means between groups. P values less than 0.05 were considered statistically significant.

## Results

**Figure 1:**
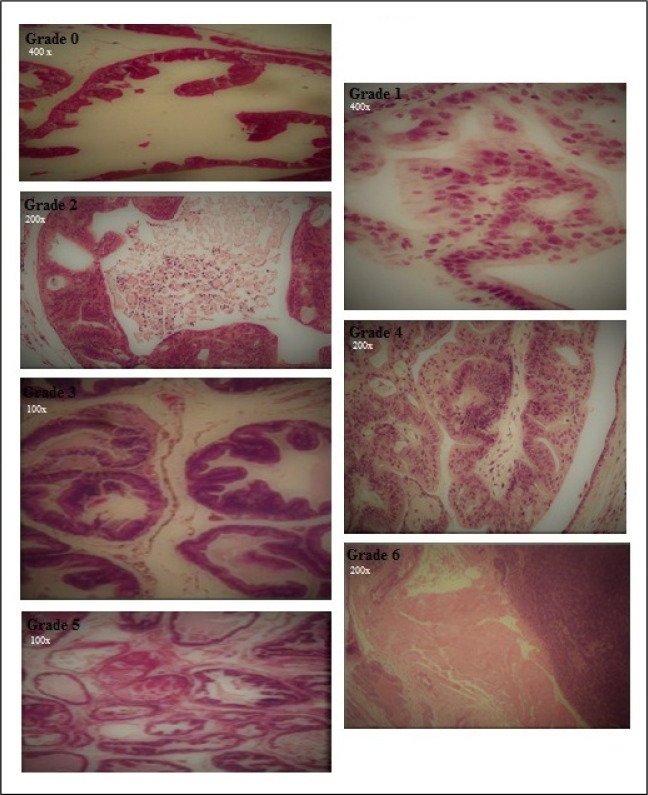
The original microscopic images for histology results of prostate. Grade 0: Normal epithelium. Grade 1: hyperplasia; Simple flat lesions. Grade 2: hyperplasia; Papillary or cribriform structures. Grade 3: hyperplasia; Papillary or cribriform lesions which protrude into lumen. Grade 4: adenoma; Lesions in which the lumen of acinus is completely filled with epithelium. Grade 5: adenoma; Lesion with a distinct epithelial mass within the lumen. Grade 6: adenocarcinoma, unwell differentiated epithelium with local invasion seems in the lesion.

**Figure 2:**
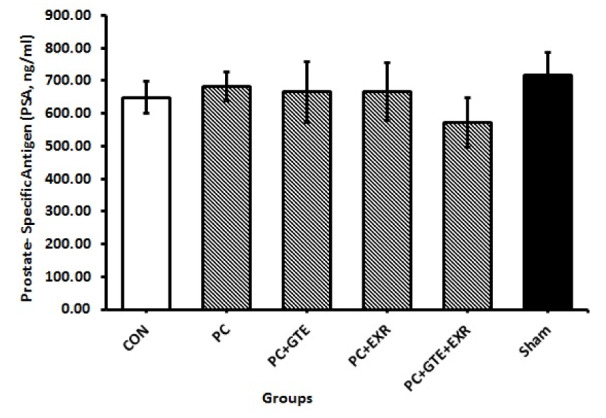
Prostate-Specific Antigen (PSA) in different groups of treated rat. CON: Healthy normal rats; PC: Rats with induced prostate cancer; PC+GTE: Rats with induced prostate cancer that received 0.1 % green tea extract. PC+EXR: Rats with induced prostate cancer that were forced to perform aerobic exercise. PC+GTE+EXR: Rats with induced prostate cancer that received 0.1% green tea extract and were forced to perform aerobic exercise simultaneously. Sham: Healthy normal rats that received an injection of physiological slaine solution as well as 1 ml of that solution as gavage. Values are mean ± standard error of eight animals. P-values less than 0.05 were considered significant.

**Figure 3:**
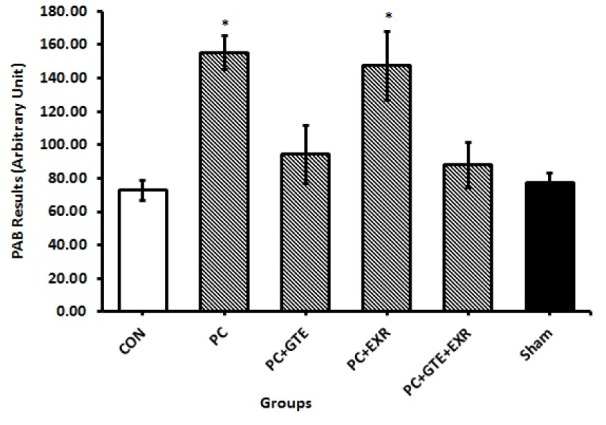
Pro-oxidant-antioxidant balance (PAB) in different groups of treated rat. CON: Healthy normal rats; PC: Rats with induced prostate cancer; PC+GTE: Rats with induced prostate cancer that received 0.1 % green tea extract. PC+EXR: Rats with induced prostate cancer that were forced to perform aerobic exercise. PC+GTE+EXR: Rats with induced prostate cancer that received 0.1 % green tea extract and were forced to perform aerobic exercise simultaneously. Sham: Healthy normal rats that received an injection of physiological saline solution as well as 1 ml of that solution as gavage. Values are mean ± standard error of eight animals. P-values less than 0.05 were considered significant. * shows P<0.05 in comparison with normal rats of CON.

No significant difference was observed in the food and fluid intake of animals during the experimental period. The amounts of body weight gain in the cancerous rats were significantly lower than controls (P<0.05). As previously mentioned to provide a prostate cancer model for this study we used a modified method to induce prostate cancer in the rats. A skilled pathologist histologically examined and scored the prostate tissues of different groups according to previously provided grading schema for prostate cancer to confirm the induction protocol. Table 1(Tab. 1) summarizes the scoring results of cancerous prostate in different groups. The mean of histological score of cancerous tissue was reduced in rat receiving HEGT and both HEGT and aerobic exercise. Figure 1(Fig. 1) also shows our original microscopic images for each specific grade. Table 2(Tab. 2) shows the amount of prostate weight in the different groups of rat. The prostate weights of cancerous rats (PC) were significantly higher than healthy controls (P < 0.05). The prostate-specific antigen (PSA) levels of different groups were presented in Figure 2(Fig. 2). There was no significant difference in the amounts of PSA levels between different groups of study (P>0.05). Figure 3(Fig. 3) shows the pro-oxidant-antioxidant balance (PAB) of differently treated rats. There was a significantly higher value of PAB for PC and PC+EXR groups in comparison with healthy controls (P<0.05). In other words, the green tea extract has prevented the increase of PAB in the UMU-induced rats receiving hydroalcoholic extract of green tea (HEGT) alone or in combination with aerobic exercise. 

## Discussion

First we established a model of prostate cancer in rat using N-Nitroso-N-methylurea (NMU). NMU has been known as an alkylating agent with a carcinogenic and mutagenic potential that transfers its methyl group to the nucleic acid bases to induce AT: GC transition mutations. It has been widely used to prepare the cancerous models of different laboratory animals. This method has been previously reported to induce prostate cancer with different efficiency (Bosland, 1992[5], 1996[6]; McCormick et al., 1998[26]; Shirai et al., 2000[36]; Arroyo-Acevedo et al., 2017[3]). To confirm the model, we conducted a histological examination for each rat. Furthermore, to correctly determine the effect of each treatment (aerobic exercise, green tea extract and combination of them) on prostate cancer, the cancerous tissues of all rats were scored in different groups. In the PC group, almost all rats clearly showed adenoma-grade hyperplasia or higher grades. To determine preventive role of green tea extract and aerobic training treatments were performed simultaneously during the process of cancer induction. Hydroalcoholic extraction of green tea alone as well as in its combination with aerobic training reduced the grade of hyperplasia in the microscopic observations. Aerobic training alone didn't show a significant change in this regard. These microscopic observations are inconsistent with the results of pro-oxidant-antioxidant balance (PAB). PAB results were only significantly higher in the PC and PC+EXR groups when compared with healthy control rats. As mentioned earlier, the PAB results represent the balance or imbalance of oxidants with antioxidants. When PAB is higher than 50, it indicates an increase in the oxidant fraction relative to the anti-oxidant fraction, and the higher this number is, the higher is the oxidant fraction.

The role of free radicals, particularly reactive oxygen species (ROS) in the development and/or the promotion of cancers should be considered to explain these results. ROS are produced in the cells as a normal reaction in response to different intracellular and extracellular changes. They play important roles in the normal physiology and metabolism of the body including cellular respiration and other immune-related functions (Gulcin, 2006[16]; Gupta-Elera et al., 2012[18]). In turn, an unusual increase in the production of reactive oxygen species or reduction of antioxidant defense provides a condition known as oxidative stress. This condition is clearly associated with numerous pathological situations, mutations and neoplastic transformation (Khandrika et al., 2009[21]). 

ROS is mainly produced during the cellular oxidation of food fuels in the aerobic organisms. Mitochondrion is an intracellular organelle considered to be the site of oxygen metabolism. When uncoupled electrons transferred to oxygen molecules the ROS will be generated. Normally 2-5 % of consumed oxygen is converted to free radicals. Exercise increases the oxidation of fuels and consequently the utilization of oxygen to provide the energy needed for the muscle activities. Thus, these events, in turn, promote the risk of free radicals production (Boveris et al., 1972[8]; Cazzola et al., 2003[10]). With this argument, we can simply explain the observed results of PAB and histology for PC+EXR group. Of course, we can't rule out the possibility of the reduction of total antioxidant capacity in this group. However, in contrast to this hypothesis, there are studies showing that exercise increases total antioxidant capacity (Berzosa et al., 2011[4]; Park and Kwak, 2016[31]). 

Tea as one of the most widely consumed drinks (about 120 ml per day) is mainly produced from the leaves of *Camellia sinensis*. The leaves of this plant are available in three forms including green tea, oolong tea and black tea depending on their fermentation degree. These three forms are also different in their antioxidant amount and components. Polyphenols (about 90 %), amino acids (about 7 %), caffeine (about 3 %) and minerals are main components of tea. The type of polyphenols also depends on the amounts of fermentation. There are more tannins in black tea, while catechins are more in green tea. In fact, green tea is considered a rich source of catechins among other dietary sources. 126 mg of catechins have been found per 100 ml of green tea (Prasanth et al., 2019[32]). The major catechins consist of epicatechin (EC), epicatechin-3-gallate (ECG), epigallocatechin (EGC), and epigallocate-chin-3-gallate (EGCG) (Lee et al., 2014[24]). The Food and Drug Administration (FDA) has reported the presence of about 71 mg of epigallocatechin-gallate per 100 ml of green tea (Rietveld and Wiseman, 2003[33]). We also determined and confirmed the presence of these major catechins in the extract we prepared from the green tea leaves using gas chromatography with detection by mass spectrometry (Saedmocheshi et al., 2019[34]). Catechins have been previously known as antioxidant compounds that reduce the production of reactive oxygen species and provide protection against cancer induction (Lambert and Elias, 2010[23]; Prasanth et al., 2019[32]). Therefore, due to the presence of catechins in green tea extract and their antioxidant benefits, the reduction of PAB and the severity of prostatic hyperplasia can be reasonable in the NMU-induced rat receiving green tea extract. Green tea leaves also contain some important elements such as selenium, zinc, and manganese in different concentrations depending on the amount of fermentation, size as well as the age of tea leaves (Cabrera et al., 2006[9]; Chacko et al., 2010[11]). Selenium is essential for the formation of the active structure of glutathione peroxidase (GPx). Zinc along with copper and manganese are also present in the structure of superoxide dismutase (SOD). These two enzymes provide a part of the body's enzymatic antioxidant defense system (Ighodaro and Akinloye, 2019[19]). Therefore, the presence of these elements in green tea extract would also favor the reduction of ROS and consequently the reduction of PAB.

Prostate-specific antigen (PSA) is a glycoprotein and chymotrypsin-like serine protease which is normally produced in the cells of the prostate gland. Its level is often elevated in the malignant cells of men with prostate cancer. Low concentrations of PSA are released into the blood. Therefore, the measurement of the PSA blood level in humans used as a laboratory test for the diagnosis of prostate cancer. In 1986 and 1994, the FDA approved the measurement of PSA as a tumor marker to monitor the progression of prostate cancer in men with already diagnosed prostate cancer. The PSA test along with the digital rectal exam is also used to evaluate asymptomatic men for prostate cancer. However, this test may have false negative or false positive results (Karr et al., 1995[20]). The specificity of this tumor marker for prostate cancer in mice, rats and other rodent is not as in human, because prostate tumors that arise in the various species may differ from human in histology and their metastatic potential (Lamb and Zhang, 2005[22]). The PSA level may also elevate in the prostatitis and benign prostatic hyperplasia (Karr et al., 1995[20]). In our induced prostate cancer model, the plasma levels of PSA didn't significantly differ from healthy controls in none of the cancerous groups. We believe that this marker is not as specific as the human for the experimental model of prostate cancer. Our finding is inconsistent with the results of some previous studies showing an increase in the plasma levels of PSA in the rat model of benign prostatic hyperplasia (Mbaka et al., 2013[25]; Arroyo-Acevedo et al., 2017[3]). The differences in the severity or progress of induced model as well as, differences in the duration and details of method such as the type of exogenous hormone used for the induction of prostate cancer may be the reasons for this inconsistency. To explain these effects, for example, we can note that the development of prostate tumor in some strains of rats are androgen responsive (Lamb and Zhang, 2005[22]).

## Conclusion

The results of our study also provided the laboratory and histological documentation for the preventive role of green tea extract in developing of prostate cancer by reducing the number of free radicals and improving the pro-oxidant-antioxidant balance.

## Author contributions

Zakaria Vahabzadeh carried out the design, supervised the study, and prepared the manuscript. Mohammadraman Molodi provided assistance in treatments of and sampling from animals, and coordinated in manuscript preparation. Bahram Nikkho as pathologist provided assistance in the histopathological examination and confirmation of prostate cancer model. Fatemeh Zamani provided assistance in the laboratory experiments. Saber Saedmocheshi, Yazdan Roshani and Sina Babanzadeh assisted in working with laboratory animals, performing treatments and sampling. Marziyeh Saghebjoo advised us for the aerobic exercise protocol of study. All authors have read and approved the content of the manuscript.

## Conflict of interest

The authors have no conflicts of interest. 

## Funding

This work, as medical students’ thesis, was supported financially by vice chancellor in research of Kurdistan University of Medical Sciences [grant numbers 97/60- 97.09.10, 96/184- 96.09.29].

## Acknowledgements

This study was entirely performed at the animal house of the Faculty of Medicine and laboratories of Cellular and Molecular Research Center of Kurdistan University of Medical Sciences. We also thank heads of the Cellular and Molecular Research Center of Kurdistan University of Medical Sciences for their valuable assistance. 

## Figures and Tables

**Table 1:**
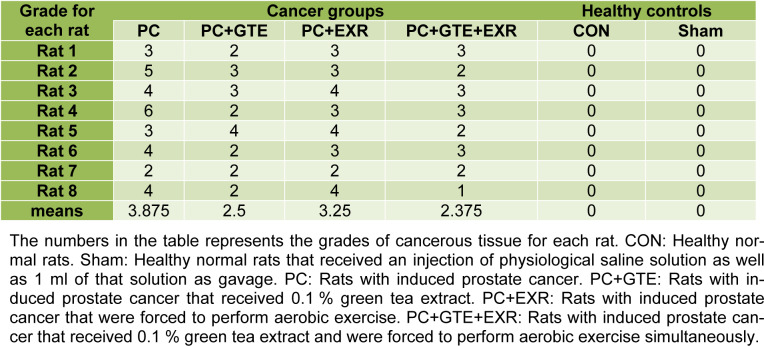
The histopathological scoring of cancerous prostate

**Table 2:**
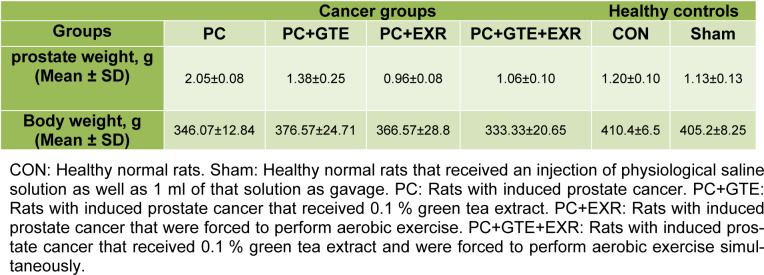
The prostate and body weight of the different groups of study
